# The Role of Gene–Gene Interaction Between *ADRA2A* and *SLC6A2* Polymorphisms in Attention System and Treatment Outcomes for Children with ADHD

**DOI:** 10.3390/children12060704

**Published:** 2025-05-29

**Authors:** Jewook Kang, Bum-Sung Choi, Bongseog Kim

**Affiliations:** 1 Department of Psychiatry, Inje University Pusan Paik Hospital, Inje University College of Medicine, Pusan 47392, Republic of Korea; jewook.kang@gmail.com; 2 Department of Psychiatry, Pusan National University Yangsan Hospital, Pusan National University School of Medicine, Yangsan 50612, Republic of Korea; 3 Research Institute for Convergence of Biomedical Science and Technology, Pusan National University Yangsan Hospital, Yangsan 50612, Republic of Korea; 4 Department of Psychiatry, Inje University Sanggye Paik Hospital, Inje University College of Medicine, Seoul 01757, Republic of Korea; kimbs328@paik.ac.kr

**Keywords:** ADHD, *ADRA2A*, *SLC6A2*, CPT, treatment outcome, gene–gene interaction, gene–dose interaction

## Abstract

Background and Objectives: Most genetic studies have focused on catecholamine system genes to identify etiology in attention-deficit/hyperactivity disorder (ADHD), and there is growing evidence that the interaction of several genes may synergistically or antagonistically affect disease outcomes. We investigated the interaction between the alpha-2 adrenergic receptor (*ADRA2A*) and its transporter (*SLC6A2*) to determine the etiology and treatment outcomes for ADHD. Materials and Methods: Children with ADHD (age 8.3 ± 2.0 y, 72 boys and 11 girls) were assessed using the Kiddie Schedule for Affective Disorders-Present and Lifetime (K-SASD-PL), ADHD rating scale-IV (ARS), Clinical Global Impressions-Improvement (CGI-I), and Clinical Global Impressions-Severity (CGI-S) scales. Neuropsychological assessments were performed using a continuous performance test (CPT). Methylphenidate was titrated based on the CGI-I and CGI-S scales for 8 weeks. We assessed two polymorphisms, *ADRA2A* rs553668 and *SLC6A2* rs998424, for their association with disease outcomes. Results: The *ADRA2A* polymorphism had a significant effect on visual/auditory commission errors in the CPT. The CC genotype for *ADRA2A* combined with the GG genotype for *SLC6A2* showed more commission errors than the other combinations of genotypes. Treatment outcome assessment using the CGI-S showed that the *SLC6A2* GG genotype had more favorable treatment outcome (*p* < 0.05) and significant gene × dose interaction on ARS score across 8 weeks (*p* < 0.01). Conclusions: Our findings provide preliminary evidence for the effect of *ADR2A* and *SLC6A2* gene–gene interactions on the attention system and treatment response in children with ADHD. Although these findings require future replication, our study contributes to the understanding of the genetic basis of ADHD.

## 1. Introduction

Attention-deficit/hyperactivity disorder (ADHD) is a common neurodevelopmental disorder that affects approximately 3–6% of school-aged children [1]. The causes of ADHD are unknown; however, ADHD is considered a multifactorial (complex) neurodevelopmental disorder. Compelling evidence has suggested the involvement of genetic factors [2]. Because heritability is high in ADHD, a large number of studies on different candidate genes for ADHD have been published, most of which have focused on genes involved in the dopaminergic neurotransmission system, because of the dopaminergic action of psychostimulant medication [3]. The noradrenergic and serotonergic systems may also be attractive candidates for the etiology of ADHD [4]. Previous neurobiological and pharmacological studies have suggested that the dysregulation of the central noradrenergic system may be involved in the pathophysiology of ADHD [5,6]. According to Arnsten’s hypothesis, alpha-2-adrenergic receptors play a major role in working memory or focused attention, as well as deficits in inhibitory control, which are known as the core deficits of ADHD [7,8]. More specifically, the alpha-2A-adrenergic receptor gene (*ADRA2A*), located on chromosome 10q24-26, has been considered an attractive candidate in genetic studies of ADHD [9]. Two major polymorphisms have been investigated: a -1291 C>G single nucleotide polymorphism (SNP) in the promoter region (rs1800544, creating an MspI site) [10] and a C>T polymorphism in the 3′ untranslated region (3′-UTR), creating a DraI site (rs553668) [11]. Studies have reported that the G allele homozygote at the MspI site is associated with higher inattention or combined symptom scores [12,13], and lower response time variability on the continuous performance test (CPT) [9]. Regarding the DraI polymorphism, the T allele has been linked to inattentive and hyperactive-impulsive symptoms [14], while the C allele has also been reported as a risk allele associated with increased response time variability [9,15].

Given their joint involvement in noradrenergic signaling, *ADRA2A* and *SLC6A2* may functionally interact to modulate synaptic norepinephrine availability. *ADRA2A* encodes presynaptic inhibitory autoreceptors that regulate norepinephrine release, while *SLC6A2* encodes the transporter responsible for norepinephrine reuptake [16,17,18,19]. Several *SLC6A2* polymorphisms, such as rs3785157, rs998424, rs11568324, rs28386840 (-3081 A/T), and G1287A (rs5569), have been evaluated for their association with ADHD [4,20,21,22]. Although findings have been inconsistent across studies [3,23,24], some evidence supports preferential transmission of specific alleles (e.g., the C allele of G1287A and the T allele of rs3785157) to ADHD probands [25]. Furthermore, the G/G genotype at rs5569 has been associated with better performance on CPT measures, suggesting a potential protective effect on attention [26]. In addition, pharmacogenetic studies have revealed that alleles G and T for the G1287A and T alleles at the -3081 (A/T) site are associated with a better response to methylphenidate [26,27]. Given the growing evidence linking norepinephrine system genes to ADHD, gene–gene interactions may play a critical role in shaping disease phenotypes. Gene variants may affect disease outcomes by acting synergistically or antagonistically; thus, their combined effect is an important aspect of disease etiology [28]. While previous studies have reported interaction effects between dopamine receptor genes and their transporters on ADHD symptomatology [29,30], little is known about norepinephrine system gene interactions.

To the best of our knowledge, few studies have been designed to ascertain the possible gene–gene interactions of the norepinephrine system. The selection of rs553668 and rs998424 polymorphisms for analysis in the present study was based on their putative roles in noradrenergic signaling, a neurotransmitter system implicated in the pathophysiology of attention-deficit/hyperactivity disorder (ADHD). Given the complementary roles of *ADRA2A* and *SLC6A2* in norepinephrine regulation, the combined analysis of these two polymorphisms may offer insight into the genetic mechanisms underlying ADHD and help clarify variability in cognitive performance and treatment response. This study aimed to examine the effect of the interaction between *ADRA2A* and *SLC6A2* polymorphisms on attention systems in ADHD. Differences in the clinical response to methylphenidate according to the genotype were also examined.

## 2. Materials and Methods

A total of 92 children diagnosed with ADHD were initially recruited for this study. Of these, nine participants were excluded for the following reasons: three withdrew consent before baseline assessment, two failed to complete the minimum treatment period of 4 weeks, and four had missing or incomplete genetic or neuropsychological data. As a result, the final sample included 83 children with ADHD (72 boys and 11 girls), who completed both the genetic analysis and neurocognitive assessments. These participants and their parents were recruited from the Department of Child and Adolescent Psychiatry at seven university-affiliated hospitals across Korea. The children selected for the ADHD group were those who (1) were 6–12 years old; (2) were diagnosed as having ADHD according to semi-structured interview; (3) scored > 25 points on the pretreatment ADHD Rating Scale (ARS); (4) had an IQ score > 80, based on the Korean version of Wechsler’s Intelligence Scale for Children; and (5) had no history of exposure to antipsychotic medication, or had <1 month exposure within 6 months at the initial visit.

Written informed consent was obtained from the parents of all children who participated in the study, which was approved by the institutional review board of Seoul National University Hospital (IRB No H-0808-021-253, 24 October 2008).

All participants were drug-naïve at the time of recruitment and were administered methylphenidate CD (MPH) for 8 weeks. Methylphenidate CD doses were administered according to weight. The starting dose was 10 mg/day for body weight < 20 kg, and 20 mg/day for body weight ≥ 20 kg. The doses were increased until a sufficient therapeutic effect on the Clinical Global Impressions (CGI) score was reached. The clinician titrated the dose upward if the CGI was >3 points and there were no intolerable side effects. If the CGI score was <2 points with tolerable side effects, the dosage was maintained. If the patient experienced any intolerable side effects, the dose was decreased. We adjusted the doses at visits 3 (study week 4) and 4 (study week 6).

The Kiddie Schedule for Affective Disorders-Present and Lifetime (K-SADS-PL) was used to diagnose ADHD. This is a semi-structured interview tool designed to evaluate the severity of ADHD symptoms and the 32 different psychiatric disorders included in the Diagnostic and Statistical Manual of Mental Disorders-IV [31]. The Korean version of the K-SADS-PL was translated, and its validity and reliability for ADHD, tic disorder, and oppositional defiant disorder were demonstrated by Kim et al. [32]. The ARS [33] and CGI were used to measure symptom severity, treatment response, and treatment efficacy. A Computerized Continuous Performance Test (CPT) [34,35] was used to measure inattention, impulsivity, and sustained attention deficits in children with ADHD.

Response criteria were defined based on the Clinical Global Impressions-improvement (CGI-I), Clinical Global Impressions-severity (CGI-S), and ARS scores at baseline and 8 weeks after MPH treatment and the dichotomous response categories, good responder vs. poor responder, were determined. Based on a change in ARS total score, a good responder had an improvement of ≥50% compared with the baseline ARS score. A second criterion was defined by the CGI-I score; a good responder scored 1 or 2 points after MPH treatment, whereas a poor responder had a CGI-I score in the range of 3–7 points [35]. A third criterion was defined by the CGI-S score after MPH treatment; a good responder scored 1 or 2, whereas a poor responder scored 3–7. As a fourth criterion, a good responder satisfies all aforementioned criteria. Kim et al. have previously applied these methods [36]. A board-certified child and adolescent psychiatrist conducted all clinical evaluations to ensure diagnostic accuracy and consistency.

Genomic DNA was extracted from peripheral blood samples collected from all participants. Genotyping was performed using a single-base primer extension assay with an ABI PRISM SNaPshot Multiplex kit (ABI, Foster City, CA, USA) according to the manufacturer’s recommendations. Briefly, the genomic DNA flanking the SNP of interest was amplified by polymerase chain reaction (PCR). Primer sequences were 5′-GGC CAC CCC TAA TCA CTA and 5′-CCA GTT GCT GGT GAA AAC for *ADRA2A* polymorphism rs553668, and 5′-ACC TTC AGC ACT TTC CTT CT and 5′-GAA GAC TAC TGG GCC TAA CC for *SLC6A2* polymorphism rs998424. PCR was performed in a 10 μL reaction volume, containing 10 ng of genomic DNA, 0.5 pM of each oligonucleotide primer, 1 μL of 10X PCR buffer, dNTPs (2.5 mM each), and 0.25 U i-StarTaq DNA Polymerase (iNtRON Biotechnology, Sungnam, Kyungki-Do, Republic of Korea). Thermocycling conditions were: 10 min at 95 °C, followed by 35 cycles of 95 °C for 30 s, Tm °C for 1 min, 72 °C for 1 min, and followed by 1 cycle of 72 °C for 10 min. After amplification, the PCR products were treated with 1 U each of shrimp alkaline phosphatase (SAP) (USB Corporation, Cleveland, OH, USA) and exonuclease I (USB Corporation, Cleveland, OH, USA) at 37 °C for 75 min and 72 °C for 15 min to purify the amplified products. One microliter of the purified amplification product was added to a SNaPshot Multiplex Ready reaction mixture containing 0.15 pmol of genotyping primer (5′-CCS ACT CTC TCT CTC TTT TT for *ADRA2A*, 5′-TGC ATA ACC ATG GTG AGT AG for *SLC6A2*) for primer extension reaction. The primer extension reaction was carried out for 25 cycles of 96 °C for 10 s, 50 °C for 5 s, and 60 °C for 30 s. The reaction products were treated with 1 U of SAP at 37 °C for 1 h and 72 °C for 15 min to remove excess fluorescent dye terminators. One microliter of the final reaction samples containing the extension products was added to 9 μL of Hi-Di formamide (ABI, Foster City, CA, USA). The mixture was incubated at 95 °C for 5 min, followed by 5 min on ice and then analyzed using electrophoresis in an ABI Prism 3730xl DNA analyzer. Analysis was carried out using GeneMapper software (version 4.0; Applied Biosystems, Waltham, MA, USA).

The allele frequencies were estimated by counting, and the Hardy–Weinberg equilibrium was calculated based on these allele frequencies, using the chi-square test.

Group differences in the clinical variables involving continuous data were computed using an independent two-sample *t*-test or one-way analysis of variance. Non-parametric tests, including the Mann–Whitney U test and Kruskal–Wallis test for continuous variables, were performed. Between-group comparisons involving categorical data were performed using the chi-square test or Fisher’s exact test. We also used analysis of variance with post hoc Bonferroni tests to compare CPT scores according to genotype.

To assess treatment outcomes, a mixed model analysis of covariance was used to analyze the main effects of MPH treatment, polymorphisms, and their interactions. The dependent variable included the ARS score (weeks 2, 4, 6, and 8), and the fixed factors were genes, MPH dose, gene × gene interaction, and gene × dose interaction. Two categorical groups (good responders vs. poor responders) according to CGI and ARS score changes were analyzed using the chi-square test. All tests were two-tailed, and significance was defined as an alpha value < 0.05.

## 3. Results

Table 1 shows the demographic characteristics according to *SLC6A2* and *ADRA2A* genotypes. The distribution of the genotypes for *SLC6A2* rs998424 was in agreement with the expected Hardy–Weinberg equilibrium (*p* > 0.05).

**Figure 1:**
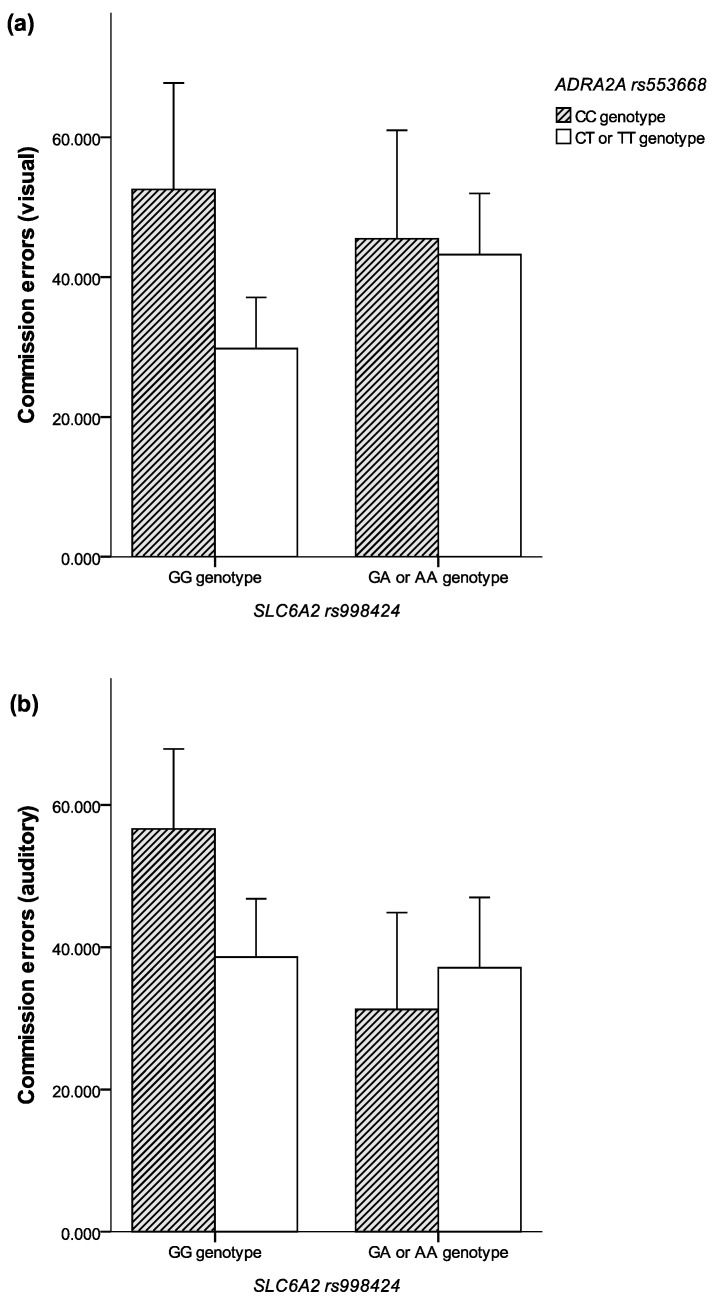
Effect of genotype interactions on commission errors. (**a**) Mean commission errors according to ADRA2A genotype (rs1800544). (**b**) Mean commission errors according to SLC6A2 genotype (rs5569).

Table 2 demonstrates the interaction effect between *ADRA2A* rs553668 and *SLC6A2* rs998424 genotypes on CPT performance. Four genotype combination groups were analyzed: (1) *ADRA2A* CC + *SLC6A2* GG, (2) *ADRA2A* CC + *SLC6A2* GA/AA, (3) *ADRA2A* CT/TT + *SLC6A2* GG, and (4) *ADRA2A* CT/TT + *SLC6A2* GA/AA. The Kruskal–Wallis test revealed significant differences across groups in visual commission errors (χ^2^ = 11.03, *p* = 0.01) and auditory commission errors (χ^2^ = 9.17, *p* = 0.03). Participants in the *ADRA2A* CC + *SLC6A2* GG group exhibited the highest number of commission errors in both modalities, indicating a possible synergistic interaction effect between the two genotypes on response inhibition. No significant interaction effects were found for omission errors, response time, or response time variability in either visual or auditory CPT conditions. These findings suggest that the combination of *ADRA2A* and *SLC6A2* risk genotypes may specifically impair inhibitory control mechanisms rather than general attention or processing speed. The effect of genotype interactions on commission errors is shown in Figure 1.

We also analyzed treatment outcomes according to genotypes and MPH dose. A potential interaction between genotype and MPH dose on the reduction in ADHD symptoms as measured by the ARS total score was observed (F_(1, 294)_ = 4.144. *p* = 0.043) (Table 3). Specifically, participants with the *SLC6A2* GG genotype exhibited a clear linear improvement in ADHD symptoms as the dose increased, suggesting a dose-dependent therapeutic effect. In contrast, those with the GA or AA genotypes showed minimal or inconsistent symptom changes across different dose levels. In addition, a three-way interaction between dose × *ADRA2A* × *SLC6A2* was statistically significant (F_(3, 294)_ = 7.503, *p* < 0.001), indicating that children carrying both risk genotypes may require tailored dosing strategies.

The association between the *SLC6A24* genotype and clinical response to MPH treatment, as measured by the CGI-S scale, is shown in Table 4. Among those with the GG genotype, 46.5% were classified as good responders, compared to only 21.6% of individuals with GA or AA genotypes (*χ*^2^ = 5.794, *p* = 0.02).

## 4. Discussion

The aim of the present study was to evaluate the effects of gene–gene interaction on attention performance in participants with ADHD. In summary, we found significant main and interaction effects of *ADRA2A* rs553668 and *SLC6A2* rs9984242 polymorphisms on CPT performance, suggesting a potential synergistic relationship between these genes in the modulation of attentional processes.

Several candidate gene studies have reported an association between *ADRA2A* polymorphisms and ADHD. For instance, Cho et al. found that participants homozygous for the C allele (C/C genotype) showed a trend toward a higher mean T-score with respect to the response time variability profiles of the CPT, but no significant differences were observed for commission errors [9]. Similarly, recent studies have shown that the T allele may contribute to fluctuations in attentional performance, supporting the functional relevance of this polymorphism in prefrontal cognitive processes [37]. Additionally, possible gene–gene interaction effects were observed between *ADRA2A* rs553668 and *BDNF* in CPT commission errors [38]. Another study reported that catechol-O-methyltransferase and monoamine oxidase A potentially predict the intelligence of participants with ADHD [39].

Our findings provide possible evidence of epistasis in genetic polymorphisms, that is, a significant synergic effect might exist on the phenotype of ADHD, and it could be assumed that there might be gene–gene interactions between adrenergic receptors and their transporter genes.

Stimulant medication has an effect not only on the dopamine transporter but also on blocking reuptake at the noradrenaline transporter [16]. Regarding treatment outcomes, our results revealed a main effect of the *SLC6A2* rs998424 genotype and a significant gene × dose interaction in predicting ADHD symptom improvement during MPH treatment. Children with the GG genotype showed a dose-dependent reduction in the ARS scores, with greater improvements at higher doses. These findings align with those of previous reports, suggesting that *SLC6A2* variants may influence stimulant responses, although prior findings have been inconsistent [26,37,40,41].

Moreover, gene × dose interactions may have a potentially greater impact than the genotype effect alone [42]. We also analyzed treatment outcomes according to genotype and MPH dose. Our data further showed that the *SLC6A2* rs998424 G/G genotype was associated with greater symptom reduction on ARS total scores compared to GA or AA genotypes. Additionally, CGI-S-based response analysis revealed that 46.5% of individuals with the *SLC6A2* rs998424 G/G genotype showed good clinical response, compared to only 21.6% with GA or AA genotypes. Although further replication is necessary, our findings suggest that specific genotypes may moderate dose–response relationships depending on treatment context. According to recent meta-analytic findings, certain polymorphisms in *SLC6A2*, such as rs28386840, may influence individual variability in response to MPH treatment in patients with ADHD [43]. Thus, accounting for gene effects and gene × dose interactions is essential in future ADHD pharmacogenetic research.

This study has several limitations. Our study assessed short-term outcomes, which are incomplete assessments of treatment responses. Long-term outcomes may be clinically relevant. In addition, the possible effects of medication on gene expression in participants with ADHD and previous exposure to MPH are unknown. To avoid this, we designed a washout period (approximately 2 weeks) for the participants included in the study. This study had no placebo-controlled group or open-label design. The inclusion of a placebo group was not feasible due to ethical concerns regarding withholding effective treatment from children with significant impairment. Practical challenges, such as recruitment and adherence, further limited the use of a randomized placebo-controlled protocol. Medication schedules allowed flexible dosing, rather than fixed dosing. Since prior studies have highlighted that ADHD symptoms often follow a linear or curvilinear dose–response curve [44,45,46], medication doses might have been lower than the optimal benefit. This may have contributed to the increase in type II errors. However, the investigators participating in our study were trained in child psychiatry and had good inter-rater reliability. The *SLC6A2* rs998424 GG genotype showed a linear relationship with MPH dosing, which is in good agreement with prior genetic studies. Another possible limitation is that the candidate genes in our study were not major genes identified in ADHD genetic studies. Because of the small sample size of our study, the results must be replicated with a large sample size.

## 5. Conclusions

The novelty of this study lies in its investigation of the gene–gene interaction between *ADRA2A* and *SLC6A2* polymorphisms in relation to both attentional performance and treatment outcomes in children with ADHD. While prior research has examined these genes individually, few studies have explored their potential synergistic effects on endophenotypic markers such as commission errors and response time variability in continuous performance tasks. Furthermore, this study is among the first to evaluate the interaction between genetic variants and MPH dosage (gene × dose effect), demonstrating that specific genotypes (e.g., of *SLC6A2GG*) may be predictive of better treatment responses. These findings contribute novel insights into the field of ADHD pharmacogenetics and underscore the importance of considering interactive genetic effects, rather than single-gene associations, when assessing treatment efficacy.

## Figures and Tables

**Table 1:** Demographic and clinical characteristics according to *SLC6A2* and *ADRA2A* genotypes.

	11 *	12 and 22 *	F or χ^2^	*p*
*SLC6A2* rs998424 (N)	45	38		
Age (y, mean ± SD)	8.69 ± 2.07	8.89 ± 1.96	0.49	0.64 ^b^
Sex				
Male/female	37/8	35/3	1.75	0.214 ^a^
Weight (kg, mean ± SD)	34.14 ± 13.11	38.53 ± 1.80	0.00	0.13 ^b^
Subtype			1.77	0.503 ^a^
ADHD-C	29	29		
ADHD-I	15	8		
ADHD-H	1	1		
*ADRA2A* rs553668 (N)	26	57		
Age (y, mean ± SD)	9.00 ± 1.8	8.68 ± 2.1	0.30	0.51 ^b^
Sex				
Male/female	24/2	48/9	1.02	0.49 ^a^
Weight (kg, mean ± SD)	38.59 ± 13.7	35.05 ± 12.0	0.79	0.38 ^b^
Subtype			0.71	0.90 ^a^
ADHD-C	18	40		
ADHD-I	8	15		
ADHD-H	0	1		

^a^ Statistical significance test using the chi-square test of Fisher’s exact test, ^b^ statistical significance test using *t*-test; * 1: Major allele (G for rs998424, C for rs553668), 2: minor allele (A for rs998424, T for rs553668). ADHD-C: combined type; ADHD-I: inattentive type; ADHD-H: hyperactive type.

**Table 2:** Interaction effect between *ADRA2A* and *SLC6A2* genotypes on CPT.

Genotypes (N)	1 ^b^ 13	2 ^b^ 12	3 ^b^ 30	4 ^b^ 24	*χ* ^2^	*p* ^a^
*Visual CPT* ^c^						
Omission Error	3.00 (0.00–32.00)	1.00 (0.00–11.00)	2.50 (0.00–30.00)	3.50 (0.00–25.00)	5.12	0.16
Commission Error	16.00 (2.00–46.00)	11.00 (0.00–42.00)	5.00 (0.00–30.00)	9.50 (1.00–41.00)	11.03	0.01
Response Time	543.70 (325.40–959.50)	482.80 (329.70–714.90)	567.90 (368.90–868.40)	510.05 (363.30–836.10)	2.57	0.46
Response Time variability	197.20 (64.70–595.50)	181.55 (60.30–316.50)	175.95 (71.80–356.70)	194.30 (87.30–632.00)	3.52	0.32
*Auditory CPT* ^c^						
Omission Error	2.00 (0.00–27.00)	0.50 (0.00–12.00)	1.00 (0.00–18.00)	1.00 (0.00–75.00)	3.78	0.29
Commission Error	8.00 (1.00–36.00)	1.00 (0.00–9.00)	3.00 (0.00–7.00)	2.50 (0.00–55.00)	9.17	0.03
Response Time	556.10 (411.40–896.20)	653.20 (448.60–914.10)	701.05 (387.10–972.80)	700.00 (0.00–1227.90)	3.12	0.37
Response Time variability	243.40 (144.90–580.40)	188.35 (88.00–329.90)	213.45 (87.70–483.90)	204.20 (70.80–783.50)	4.07	0.25

^a^ Statistical significance was assessed using the Kruskal–Wallis test. ^b^ Genotypes: *ADRA2A*_*SLC6A2*, 1: CC+GG; 2: CC/GA+AA; 3: CT+TT/GG; 4: CT+TT/GA+AA. ^c^ Data are expressed as median (25th to 75th percentile).

**Table 3:** Gene and gene × dose interaction effects on ARS total scores changes.

	df	F	*p* Value
Main effects			
MPH dose (Dose)	(6, 294)	5.797	<0.001
*ADRA2A* rs553668	(1, 294)	2.456	0.118
*SLC6A2* rs998424	(1, 294)	4.129	0.043
Interaction effect			
Dose × *ADRA2A*	(4, 294)	0.411	0.801
Dose × *SLC6A2*	(4, 294)	3.317	0.011
*ADRA2A* × *SLC6A2*	(1, 294)	0.812	0.368
Dose × *ADRA2A* × *SLC6A2*	(3, 294)	7.503	<0.001

Dependent variable: ARS total score. MPH, methylphenidate.

**Table 4:** Association between *SLC6A2* genotype and response to MPH treatment according to the CGI -S score.

*SLC6A2* rs998424	Response to MPH by CGI-S (% Within *SLCA6A* Genotype)		Total (%)	OR	95% CI		*p*
	Poor (CGI-S Score: 3–7)	Good (CGI-S Score: 1–2)			Lower	Upper	
G/G	23 (53.5%)	20 (46.5%)	43 (53.8%)	3.152	1.176	5.045	0.020 ^a^
G/A or A/A	29 (78.4%)	8 (21.6%)	37 (46.3%)				
Total	52 (65.0%)	28 (35.0%)	80				

*χ*^2^ or Fisher’s exact test was used for association. ^a^ Pearson *χ*^2^ = 5.794 by Fisher’s exact test. OR, odds ratio; CI, confidence interval; MPH, methylphenidate; CGI-S, Clinical Global Impressions-severity.

## Data Availability

The original contributions presented in this study are included in the article. Further inquiries can be directed to the corresponding author.
